# Gang membership and sexual violence: associations with childhood maltreatment and psychiatric morbidity

**DOI:** 10.1192/bjp.2020.69

**Published:** 2020-10

**Authors:** Jeremy Coid, Rafael A. González, Constantinos Kallis, Yamin Zhang, YuanYuan Liu, Jane Wood, Zara Quigg, Simone Ullrich

**Affiliations:** 1 Brain Research Center and Mental Health Center, West China Hospital of Sichuan University, China; and Wolfson Institute of Preventive Medicine, Queen Mary University of London, UK; 2 Centre for Psychiatry, Imperial College London, UK; 3 Wolfson Institute of Preventive Medicine, Queen Mary University of London, UK; 4 Brain Research Center and Mental Health Center, West China Hospital of Sichuan University, China; 5 West China School of Public Health, Sichuan University, China; 6 School of Psychology, University of Kent, UK; 7 Public Health Institute, Liverpool John Moores University, UK

**Keywords:** Epidemiology, psychotic disorders, childhood experience, alcohol disorders, drugs of dependence disorders

## Abstract

**Background:**

Gang members engage in many high-risk sexual activities that may be associated with psychiatric morbidity. Victim-focused research finds high prevalence of sexual violence towards women affiliated with gangs.

**Aims:**

To investigate associations between childhood maltreatment and psychiatric morbidity on coercive and high-risk sexual behaviour among gang members.

**Method:**

Cross-sectional survey of 4665 men 18–34 years in Great Britain using random location sampling. The survey oversampled men from areas with high levels of violence and gang membership. Participants completed questionnaires covering violent and sexual behaviours, experiences of childhood disadvantage and trauma, and psychiatric diagnoses using standardised instruments.

**Results:**

Antisocial men and gang members had high levels of sexual violence and multiple risk behaviours for sexually transmitted infections, childhood maltreatment and mental disorders, including addictions. Physical, sexual and emotional trauma were strongly associated with adult sexual behaviour and more prevalent among gang members. Other violent behaviour, psychiatric morbidity and addictions accounted for high-risk and compulsive sexual behaviours among gang members but not antisocial men. Gang members showed precursors before age 15 years of adult preference for coercive rather than consenting sexual behaviour.

**Conclusions:**

Gang members show inordinately high levels of childhood trauma and disadvantage, sexual and non-sexual violence, and psychiatric disorders, which are interrelated. The public health problem of sexual victimisation of affiliated women is explained by these findings. Healthcare professionals may have difficulties promoting desistance from adverse health-related behaviours among gang members whose multiple high-risk and violent sexual behaviours are associated with psychiatric morbidity, particularly addictions.

Psychiatric morbidity and violent behaviour are closely associated and inordinately prevalent among street gang members.^1^ Sexual assault and exploitation of gang-affiliated women have been underreported and rarely included in studies of gang violence but gained recognition as a serious public health problem following publication of three UK reports.^2–4^ Few gang-affiliated women report rape because of fear of violent retaliation and because neither victims nor perpetrators identify unwanted sex as rape.^2–4^ Mechanisms involved in gang-related sexual violence are not fully understood. However, sexual access through instrumental violence is one of several commodities in gangs, including money and substances.^1,5^ Sexual coercion is also among several violent behaviours shown by severely antisocial individuals where violent versatility involving different victim types, multiple heterosexual encounters, enhancement of self-esteem through dominance of sexual partners and overt homophobia reinforced by subculture and music^6^ correspond to the hypermasculine gang environment. For some, excitement from sensation-seeking through multiple forms of sexual risk-taking^7^ could be associated with sexual violence. A key research question is whether aetiological factors and early behavioural equivalents, or precursors, of adult sexual violence and high-risk behaviour are observed before or after joining a gang. Adult gang members typically show life-course persistence of early violence and conduct disorder to antisocial personality disorder (ASPD).^1,8^ Conduct disorder is strongly associated with child maltreatment and both predispose to joining a gang.^9^ Among different forms of maltreatment, sexual abuse has a specific association with adult sexual offending.^10,11^ However, there is little information on childhood sexual abuse among gang members, associations between childhood maltreatment and their adult sexual behaviour, or other psychopathological factors that may explain these behaviours in adulthood.

The aims of this study were to investigate (a) associations between childhood experiences of maltreatment and coercive and high-risk sexual behaviours in adulthood in a nationally representative sample of young men; (b) explanatory effects of psychiatric morbidity, addictive behaviours and violence in adulthood on their sexual behaviour. Because gang membership involves a highly antisocial lifestyle, to identify the specific effects of gang membership on sexual behaviour we compared gang members with young men with adult antisocial personality disorder (AAS – defined as three or more DSM-IV ASPD criteria in adulthood, irrespective of whether or not they qualified for a DSM-IV diagnosis of conduct disorder before age 15 years) but not in gangs, and with other young men.

## Method

### Data collection

This study has been previously described.^1^ The survey was carried out in 2011 based on random location sampling. Individual sampling units (census areas of 150 households) were randomly selected within British regions in proportion to their population to derive a representative sample of young men (18–34 years) from England, Scotland and Wales. There were four additional, boost surveys oversampling young Black and minority ethnic men, and those from lower social grades. Two boost surveys oversampled from output areas in locations characterised by high gang membership: the London Borough of Hackney and Glasgow East, Scotland. The same sampling principles applied to each survey type.

The self-administered questionnaire piloted in a previous survey was adapted and informed consent obtained from respondents. Participants were contacted in person by interviewers and, if agreeing to participate, completed the pencil and paper questionnaire in private and returned it to the interviewer. All participants were paid £5 for taking part in the survey and all questionnaires were anonymised.

Weights were constructed for each survey using Random Iterative Method (RIM) weighting to ensure representativity of the sample. All descriptive and subsequent statistical comparisons were based on weighted data.

The authors assert that all procedures contributing to this work comply with the ethical standards of the relevant national and institutional committees on human experimentation and with the Helsinki Declaration of 1975, as revised in 2008. All procedures involving human participants were approved by Queen Mary University of London ethics committee.

### Survey measures

The Psychosis Screening Questionnaire^12^ screened participants for psychosis when ≥3 criteria were met. The Hospital Anxiety and Depression Scale^13^ was used to define anxiety and depression based on the score of >11 in the past week. Scores >20 on the Alcohol Use Disorders Identification Test^14^ and scores of >25 on the Drug Use Identification Test^15^ were used to identify alcohol or drug dependence, respectively. The South Oaks Gambling Screen identified pathological gambling at a cut-off of >5.^16^

Questions from the Structured Clinical Interview for DSM-IV Personality Disorders Screening Questionnaire^17^ identified antisocial personality disorder (ASPD) consisting of conduct disorder and adult antisocial items. For our comparison group with gang members, adult antisocial personality disorder (AAS) only was used when three or more of seven items were present (DSM: 301.7). We used 312.8 conduct disorder item 7 ‘has forced someone into sexual activity’ before age 15 to identify a putative precursor of adult coercive or other adult sexual behaviour. We retained a categorical diagnostic measure of conduct disorder when a participant scored 3 or more on the remaining 14 items. Participants also reported whether they had first experienced sexual intercourse before 13 years.

Participants screened positive for problem pornography use when endorsing ≥3: prolonged time, spending excessive money, feeling bad about, losing relationships, seeking help, fear of and actual police involvement, due to pornography use, and concealing use.

We measured stalking using the Obsessive Relational Intrusion Scale (short form) when two behaviours, including pursuit, violation, threat or hyperintimacy had occurred more than once.^18^

### Gang membership, child maltreatment and sexual behaviour

All participants were questioned about their sexual behaviour, including close relationships, high-risk, sensation-seeking and putative compulsive sexual behaviours in adulthood. This included whether they had ten or more sexual partners in the past year, sex usually occurred when intoxicated, contraceptives used rarely/never, sex with a prostitute ten occasions or more in lifetime, sex with multiple partners during the same encounter ever, currently having sex with men, anal sex ever, been paid themselves ever for sex, and regularly visited strip/lap dancing clubs or parties ≥2 per week. They were also asked whether they had forced partners to have sex against their will in the past year and in more than half of encounters in the past year, and whether they had ever been convicted of a sexual offence.

They were asked about experiences of maltreatment and disadvantage before age 16 years including witnessing violence in the home, sexual abuse, physical abuse, neglect, being placed in care and first sexual intercourse <13 years. They were asked questions about other violence over the past 5 years, including whether they had deliberately been violent to obtain money, drugs or sex, whether they were involved in violence because they found it exciting, and whether they would become violent if disrespected.

They were asked ‘Are you currently a member of a gang?’ and included three of the five UK criminal justice agency criteria^19^ that could be captured using self-report, covering predominantly street-based individuals who see themselves as a discernible group, engage in criminal activity or violence and are in conflict with similar gangs. For inclusion in the study, gang members had to endorse gang membership and one or more of the following: serious criminal activities or convictions, involvement with friends in criminal activities or involvement in gang fights during the past 5 years. Participants were divided into three mutually exclusive groups:

(a) other men group: participants who did not screen positive for AAS and did not report gang membership;
(b) AAS group – participants screened positive for AAS but did not report gang membership;
(c) Gang member group.

### Statistical analysis

We initially compared the demographic characteristics of the other men group, those with AAS and gang members using logistic regression to identify potential confounders. Three analyses were performed comparing other men with AAS, other men with gang members, and the AAS group with gang members. Differences between other men, those with AAS and gang members with respect to childhood maltreatment, adolescent precursors of violent and sexual behaviours, and sexual behaviour in adulthood, psychiatric morbidity, addictive disorders, and other violence were established by performing logistic regression analyses in the three comparison groups. As above, the three analyses were conducted comparing other men and those with AAS, other men and gang members, and those with AAS and gang members.

Finally, we investigated whether associations between (a) gang membership, (b) violent and criminal sexual behaviour, compulsive behaviour, and HIV/sexually transmitted infection (STI) risk behaviour were explained by specific characteristics of other violent behaviour (instrumental, for excitement, because of disrespect), psychiatric morbidity (depression, anxiety, psychosis) and addictive behaviour (alcohol and drug dependence, problem gambling) using an explanatory statistical model used in a previous investigation with this sample.^1^ Potential explanatory variables were first identified by testing their association with (a) gang membership and violent/criminal sexual behaviour, compulsive behaviour and HIV/STI risk factors, and (b) characteristics of other violent behaviour, psychiatric morbidity, addictive behaviours. Only if both associations were significant at an alpha level of 0.05 were variables selected and then entered in an adjusted model, with group membership as the independent variable and characteristics of violence, psychiatric morbidity or addictive behaviour as the dependent variable.

We examined the percentage reduction in the baseline odds of each sexual behaviour after adding each of the potentially explanatory variables into the following equation:
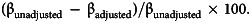


The percentage change was calculated based on beta scores, from models on the logit scale (i.e. not exponentiated). In a final model, all explanatory variables were entered simultaneously. Comparisons between baseline-adjusted and fully adjusted coefficients were used to estimate the extent to which the association between group membership and sexual behaviour was accounted for by the explanatory variable.

To control for differences between samples, survey type (i.e. random or boost) was included as a covariate in all analyses using the Huber–White sandwich approach. All models were adjusted for age, being single, employment status, being non-UK born and ethnicity. We also used robust standard error to account for correlations between survey areas because of clustering within postal codes. An alpha level of 0.05 was adopted throughout. All analyses were performed in Stata Version 12 (StataCorp, College Station, Texas. USA).

## Results

### Demography and sampling

The weighted sample included 4665 men who were 18–34 years of age: 1822 (39.1%) from the main survey; 969 (20.8%) from the ethnic minority sample; 555 (11.9%) men from lower social classes; 624 (13.4%) from Hackney; and 694 (14.9%) from Glasgow East. Of the total sample, 108 (2.3%) reported current gang membership, 678 (14.5%) ≥3 were in the AAS group, and there were 3879 (83.2%) other men.

Table 1 shows that AAS men were older on average than those in the other men group, more were unemployed, but fewer originated from the Indian subcontinent or lived in Hackney. Gang members were younger than those in the other men group, less likely to be single or non-UK born, but more likely to be unemployed, Black or from the Indian subcontinent. Compared with the AAS men group, gang members were also more likely to be younger, less likely to be single and non-UK born, and more likely to be Black and from the Indian subcontinent.

**Table 1:** Demographic characteristics of gang members, men with adult antisocial personality disorder (AAS) and other men^a^

Characteristic	Other men group	AAS group	Gang member	AAS group compared with other men group		Gang members compared with other men group		Gang members compared with AAS group	
				AOR	95% CI	AOR	95% CI	AOR	95% CI
Non-UK born, *n* (%)	586 (14.8)	35 (6.9)	5 (4.6)	0.79	0.51–1.23	0.15***	0.06–0.39	0.19***	0.07–0.52
Single, *n* (%)	2494 (62.5)	312 (60.3)	61 (57.7)	1	0.78–1.27	0.43**	0.26–0.72	0.43**	0.25–0.76
Unemployed, *n* (%)	1416 (35.9)	254 (50.1)	51 (50.4)	1.93***	1.54–2.42	1.98**	1.22–3.21	1.03	0.61–1.72
Ethnicity, *n* (%)									
White (reference)	2499 (62.0)	442 (84.9)	37 (34.1)	ref	ref	ref	ref	ref	ref
Black	554 (13.8)	54 (10.4)	53 (49.4)	0.65	0.4–1.05	10.64***	6–18.88	16.49***	8.24–33.02
Indian subcontinent	908 (22.5)	22 (4.3)	16 (15.3)	0.17**	0.09–0.35	2.62**	1.28–5.37	15.15***	5.99–38.33
Other	68 (1.7)	2 (0.4)	1 (1.2)	0.17	0.05–0.63	2.35	0.53–10.46	13.61**	1.99–93.11
Survey type, *n* (%)									
Main (reference)	1550 (38.4)	253 (48.7)	19 (17.8)	ref	ref	ref	ref	ref	ref
Ethnic minorities	910 (22.5)	51 (9.8)	8 (7.9)	0.92	0.48–1.78	0.28*	0.1–0.76	0.30*	0.1–0.94
Lower social classes	457 (11.3)	83 (15.9)	16 (14.6)	1	0.73–1.37	2.38*	1.08–5.22	2.38*	1.06–5.36
London, Hackney	540 (13.4)	30 (5.8)	54 (49.9)	0.49**	0.29–0.8	4.20***	1.93–9.15	8.65***	3.67–20.41
Glasgow East	580 (14.4)	103 (19.7)	11 (9.8)	0.73	0.53–1.03	2.44	0.86–6.92	3.32*	1.14–9.69
Age, years: mean (s.d.)	26.2 (5.0)	27.1 (5.0)	25.1 (5.3)	1.05***	1.02–1.07	0.94*	0.89–0.99	0.90***	0.85–0.95

AOR, adjusted odds ratio; ref, reference.

a. Each covariate adjusted for other covariates.

*P < 0.05; **P < 0.01; ***P < 0.001. *n* values may differ due to missing data. See supplementary Table 4 for further information.

### Sexual behaviour, childhood maltreatment and psychopathology

Table 2 shows analyses of reported experiences of child maltreatment, adult sexual behaviour, other associated violence, psychiatric morbidity and addictions, comparing the gang members, the AAS men group and the other men group. The data show a marked gradient with each category infrequent among the other men group but increasing progressively from the AAS men group to gang members. This gradient was confirmed for all outcomes (*P* < 0.001) including addictions. However, the gradient was less apparent for several adult sexual behaviours where there were fewer significant differences between the AAS men and other men groups, but with extreme differences because of higher prevalence among gang members.

**Table 2:** Adult sexual behaviour, psychiatric morbidity, addictive, adult violent behaviour and gang membership (*n* = 4665)^a^

Measure	Other men group *n* = 3879 (83.2%)		AAS group *n* = 678 (14.5%)		Gang member group *n* = 108 (2.3%)		Men with AAS compared with other group		Gang members compared with other group		Gang members compared with AAS group	
	*n*	%	*n*	%	*n*	%	AOR	95% CI	AOR	95% CI	AOR	95% CI
*Sexual behaviour*												
Violent/criminal												
Coercive sex < 15 years	14	0.3	11	2.1	43	42.3	14.75***	5.97–36.46	158.04***	71.78–347.96	10.71***	3.78–30.39
Coercive sex (past year)	337	10.1	172	27.9	67	72.4	3.36***	2.62–4.31	16.58***	9.77–28.15	4.93***	2.80–8.68
Coercive sex preference	104	3	30	6.3	29	31.6	2.37***	1.47–3.81	12.63***	6.76–23.57	5.33***	2.59–10.99
Stalking	53	1.3	28	5.5	44	42.1	4.62***	2.70–7.88	42.89***	25.15–73.14	9.29***	4.89–17.66
Conviction sex offence	0	0	1	0.2	10	9.4	–	–	–	–	–	–
Compulsive behaviours												
Strip/lap dancing clubs (≥2 weekly)	171	4.4	11	2.2	25	5.3	0.59	0.30–1.7	3.39***	1.84–6.23	5.70***	2.28–14.26
Partying (≥2 weekly)	476	12.1	66	12.9	49	46.9	1.18	0.86–1.62	5.24***	3.25–8.43	4.44***	2.60–7.57
Problem pornography use	17	0.4	14	2.7	32	30	8.73***	3.44–22.12	51.03***	25.82–100.85	5.85**	2.10–16.31
HIV/STI risk												
Contraceptives rare/never	912	26.6	180	35.3	57	56.3	1.66**	1.32–2.08	2.80***	1.80–4.38	1.69*	1.04–2.75
Anal sex	560	15.2	174	34.7	58	56.2	2.80***	2.21–3.56	9.32***	5.80–14.97	3.33***	1.99–5.55
Paid for sex	89	2.4	44	8.5	27	27.6	4.40***	2.82–6.86	10.36***	5.93–18.09	2.35**	1.22–4.54
STI (ever)	239	6.4	133	26.1	48	47.5	4.39***	3.31–5.83	13.47***	8.16–22.22	3.07***	1.77–5.32
≥10 partners past year	188	5.3	44	9.1	37	35.7	1.95**	1.29–2.96	6.63***	3.77–11.66	3.40***	1.85–6.23
Prostitutes (≥10) ever	26	0.7	11	2.1	28	26.6	3.22*	1.31–7.96	27.65***	12.96–58.99	8.58***	3.29–22.35
Sex usually intoxicated	543	15.8	201	42	68	67.1	3.56***	2.80–4.52	9.11***	5.31–15.64	2.56**	1.46–4.50
Multiple partners (same encounter)	536	14.6	199	39	57	57.6	3.44***	2.76–4.29	10.16***	6.20–16.64	2.95***	1.75–4.98
Sex with men	116	3.1	27	5.3	23	22.6	1.51	0.87–2.60	7.65***	4.22–13.88	5.07***	2.29–11.24
*Child maltreatment*												
Witnessing violence in home	419	10.4	239	46	47	43.4	6.33***	5.03–7.96	9.50***	5.79–15.59	1.5	0.90–2.50
Sexual abuse	62	1.5	32	6.2	20	18.6	4.19***	2.54–6.91	15.01***	7.94–28.37	3.58**	1.73–7.41
Physical abuse	150	3.7	103	19.7	31	29	5.64***	4.11–7.73	12.40***	7.05–21.82	2.20**	1.21–3.98
Neglect	112	2.8	82	15.7	32	30.2	7.37***	5.17–10.53	15.02***	8.32–27.13	2.04*	1.10–3.77
First intercourse <13 years	146	4.5	114	22.9	49	47.8	5.65***	4.07–7.83	14.71***	8.99–24.09	2.61**	1.52–4.47
In care	77	2	61	12	18	19.2	5.39***	3.60–8.07	8.98***	4.03–20.02	1.67	0.73–3.79
*Violent behaviour (past 5 years)*												
Instrumental violence	50	1.3	72	13.8	77	71.9	13.52***	8.48–21.57	174.00***	99.79–303.38	12.87***	6.98–23.72
Violence for excitement	102	2.5	101	19.3	58	53.6	9.43***	6.76–13.14	45.47***	26.58–77.79	4.82***	2.70–8.64
Violent if disrespect	519	12.9	266	51.2	87	81.3	7.00***	5.59–8.77	29.54***	15.20–57.42	4.22***	2.13–8.35
*Psychiatric morbidity*												
Psychosis	52	1.3	33	6.5	26	25.1	6.64***	3.96–11.15	18.23***	9.12–36.44	2.75**	1.38–5.46
Anxiety disorder	449	11.3	136	26.5	63	58.9	3.12***	2.40–4.06	10.59***	6.27–17.91	3.40***	1.95–5.93
Depressive disorder	345	8.7	65	12.5	21	19.7	1.57**	1.12–2.20	1.14	0.51–2.53	0.72	0.32–1.65
*Personality disorder*												
Conduct disorder	426	11.1	476	71.3	95	88.5	17.88***	14.33–22.30	86.67***	35.58–211.13	4.85**	1.96–11.98
Adult antisocial (AAS)	0	0.0	678	100.0	95	92.5						
Antisocial personality disorder	0	0.0	476	71.9	86	85.8						
*Addictive behaviour*												
Drug dependence	22	0.6	65	12.8	59	57.4	20.80***	11.10–38.99	196.46***	94.45–408.63	9.44***	4.77–18.71
Alcohol dependence	266	6.8	99	19.9	68	66.6	3.44***	2.54–4.65	23.27***	13.11–41.33	6.77***	3.72–12.33
Pathological gambling	60	1.7	53	10.6	57	55.7	6.99***	4.39–11.13	36.85***	21.57–62.93	5.27***	2.93–9.46

AAS, adult antisocial personality disorder; AOR, adjusted odds ratio; STI, sexually transmitted infection.

a. All weighted frequencies, percentages and estimates (AOR, 95% CI). Adjusted for age, being single, employment status, non-UK born, ethnicity and survey type.

*P < 0.05; **P < 0.01; ***P < 0.001. *n* values may differ due to missing data. See supplementary Table 4 for further information.

The three pairwise sets of analysis were then used to explore the relationships in more detail (Table 2). The AAS men group differed significantly from the other men group on all measurements except attending strip/lap dancing clubs and partying. The differences between gang members and the other men group in relation to child maltreatment, child/adolescent precursors, adult sexual behaviour, other violence, psychiatric morbidity and addictions were considerably greater, except for depression. Gang members demonstrated higher levels of all items than the AAS men group, except for depression (see also supplementary Fig. 1 available at https://doi.org/10.1192/bjp.2020.69)

### Childhood maltreatment and adult sexual behaviour

Table 3 shows associations between adverse childhood experiences (including child maltreatment) and the dependent variables of sexual behaviour in adulthood in the total sample. Witnessing violence in the home was associated with coercive adolescent sexual behaviour, problem pornography use, anal sex, STIs, sex usually intoxicated, multiple partners in same encounter. Sexual abuse was associated with adolescent coercive sex, anal sex, being paid for sex, ≥10 encounters with prostitutes. Physical abuse was associated with sex usually intoxicated, multiple partners in same encounter. Neglect was associated with adolescent coercive sex, paid for sex, STIs. First sexual experience was associated before 13 years with adolescent coercive sex, coercive sex in adulthood, stalking, attending strip/lap dancing clubs, anal sex, STIs, ≥10 partners in past year, ≥10 encounters with prostitutes, sex usually intoxicated, multiple partners in same encounter. Being placed in care was associated with attending strip/lap dancing clubs, anal sex, paid for sex and sex with men.

**Table 3:** Effects of child maltreatment and adverse experiences on adult sexual behaviour (*n* = 4664)^a^

Sexual behaviour	Witnessing violence in home		Sexual abuse		Physical abuse		Neglect		First sex <13 years		In care	
AOR	95% CI	AOR	95% CI	AOR	95% CI	AOR	95% CI	AOR	95% CI	AOR	95% CI
Violent/criminal												
Coercive sex <15 years	4.40**	1.59–12.19	4.67**	1.59–13.69	1.41	0.51–3.90	5.59***	2.64–11.83	7.64***	3.02–19.29	1.11	0.30–4.11
Adult coercive sex	1.35	0.78–2.33	1.8	0.64–5.06	1.34	0.69–2.58	0.5	0.25–1.01	2.75***	1.57–4.82	1.98	0.74–5.27
Stalking	1.62	0.92–2.84	1.55	0.71–3.35	0.82	0.43–1.55	2.32*	1.22–4.42	4.30***	2.60–7.12	1.23	0.55–2.76
Conviction sex offence	–	–	–	–	–	–	–	–	–	–	–	–
Compulsive behaviours												
Strip/lap dancing clubs (≥2 weekly)	0.61	0.31–1.18	2.06	0.88–4.80	1.67	0.77–3.64	0.82	0.41–1.66	2.16*	1.20–3.87	2.58*	1.03–6.46
Partying (≥2 weekly)	1.08	0.78–1.51	0.97	0.49–1.94	1.54	0.96–2.47	0.97	0.58–1.61	1.15	0.75–1.75	1.51	0.86–2.65
Problem pornography use	7.15***	2.54–20.10	3.41	0.90–12.99	0.55	0.15–1.98	1.33	0.60–2.95	2.32	0.92–5.85	1.03	0.34–3.14
HIV/STI risk												
Anal sex	1.49**	1.15–1.93	2.84***	1.59–5.08	1.14	0.77–1.67	1.24	0.82–1.88	1.83***	1.33–2.52	1.70*	1.10–2.63
Paid for sex	1.36	0.84–2.18	2.82**	1.33–5.98	1.52	0.82–2.80	1.87*	1.02–3.43	1.45	0.76–2.78	2.12*	1.12–4.02
STI (ever)	1.97***	1.44–2.70	1.37	0.68–2.76	1.37	0.87–2.16	1.85**	1.18–2.90	2.61***	1.82–3.73	1.23	0.70–2.16
≥10 partners past year	1.46	0.89–2.40	1.46	0.68–3.15	1.77	0.99–3.19	1.39	0.79–2.46	1.83*	1.11–3.00	1.56	0.80–3.03
Prostitutes (≥10)	1.17	0.43–3.21	3.48**	1.44–8.42	1.4	0.58–3.35	0.66	0.29–1.48	4.09**	1.79–9.35	2.5	0.75–8.27
Sex usually intoxicated	1.56***	1.22–2.00	0.75	0.41–1.40	1.88**	1.25–2.83	0.97	0.63–1.51	1.80**	1.28–2.53	1.47	0.90–2.39
Multiple partners (same encounter)	1.34*	1.05–1.72	1.11	0.61–2.02	1.71**	1.18–2.50	0.99	0.66–1.49	2.51***	1.83–3.44	1.53	0.96–2.43
Sex with men	1.76	0.96–3.23	2.27	0.97–5.29	0.7	0.37–1.31	1.12	0.59–2.13	1.03	0.52–2.04	2.99**	1.45–6.13

AOR, adjusted odds ratio; STI, sexually transmitted infection.

a. All weighted frequencies, percentages and estimates (AOR, 95% CI). Adjusted for age, being single, employment status, non-UK born, ethnicity and survey type.

*P < 0.05; **P < 0.01; ***P < 0.001.

### Explaining links between violence, psychiatric morbidity, addictions and adult sexual behaviour

We investigated whether other violence associated with gang activity, psychiatric morbidity and addictions explained the elevated rates of sexual behaviour in adulthood among the antisocial men group and gang members. Supplementary Table 1 presents the change in odds of four domains of sexual behaviour in adulthood among the antisocial men group compared with the other men group after accounting for other violence (instrumental, excitement, due to disrespect), psychiatric morbidity (psychosis, anxiety disorder, depression), addictions (drug and alcohol dependence and pathological gambling), (percentage of change in odds explained by these variables). Reductions can be seen for each of the outcomes. However, only coercive sex preference and varied sexual experiences of ≥10 partners in the past year and sex with prostitutes were no longer significant.

Comparison of the gang member group and other men group (supplementary Table 2) showed that attending strip/lap dancing clubs, partying, contraceptive use rare/never, paid for sex, STIs and >10 partners in the past year were no longer significant and explained by other violent behaviour, psychiatric morbidity and addictions.

Comparison of the gang member and antisocial men groups (supplementary Table 3) showed that higher rates of coercive sex preference, visiting strip/lap dancing clubs, contraceptive use rare/never, paid for sex, STIs, ≥10 partners in the past year, sex with prostitutes, sex usually intoxicated and multiple partners in the same encounter were substantially explained by other violent behaviour, psychiatric morbidity and addictions.

## Discussion

### Violent and high-risk sexual behaviour

We found high levels of sexual violence and multiple risk behaviours for STIs and HIV among British gang members. We observed a marked gradient in levels of these behaviours together with psychiatric morbidity, including addictions, characteristics of their non-sexual violence and childhood adverse experiences across the three groups. In general, all were more prevalent among antisocial men and gang members than among the other men group. Because most of these factors were more prevalent among gang members than the antisocial men group, our findings explain the public health problem of widespread sexual violence and exploitation of gang-affiliated women reported in victim-focused research.^2–4^

Among male-dominated gangs, female members are considered inferior, expected to be subservient and provide sex. Many perceive sexual violence as normal and inevitable.^2–4^ More than 40% of gang members in our study reported coercive sexual behaviour before age 15 years and nearly a third reported coercion during most sexual encounters during the past year. Only gang members reported convictions for a sexual offence. These findings, together with repetitive use of pornography and prostitutes, multiple sexual partners and frequent attendance at strip/lap dancing clubs indicated components of hypersexuality and compulsivity in their sexual behaviour. These findings also questioned whether, for some, violent rather than consensual sex had become preferential.^20,21^

We confirmed high rates of risk-taking behaviours previously observed in gangs including sex when intoxicated, infrequent condom use, sex with prostitutes, STIs, sex for money and group sex.^22,23^ Group sex in gangs can be part of initiation and occurs at parties where it is often unclear whether sex is consensual or coerced.^7,24–27^ Reports of same-sex behaviour were new findings. Gay identity is rarely reported and usually considered unacceptable in the hypermasculine gang environment.^28^ Nevertheless, gang members are more likely to report sex while in prison.^23^ Our finding that one in five reported sex with men requires further investigation, questioning whether predatory sexual behaviour can include younger male gang members as well as affiliated females.

### Childhood maltreatment and sexual behaviour in adulthood

We found a marked gradient in experiences of childhood maltreatment across the three groups. All forms were more prevalent among the antisocial men group and gang members. However, sexual and physical abuse, neglect, and early sexual initiation were all more common among gang members than antisocial men. When examined across the entire sample, childhood maltreatment experiences showed strongest associations with forcing sex on others before age 15 years, although these were weaker for coercive sex in adulthood. Sexual coercion before 15 years may therefore have been the precursor to equivalent behaviour in adulthood. However, it required specific situational or social environmental factors for its perpetuation into adulthood, in this case gang membership.

High-risk behaviours including anal sex, being paid for sex, sex with prostitutes and STIs were more strongly associated with child maltreatment experiences than coercive behaviour, particularly early sexual initiation and sexual abuse. These findings correspond to research showing children exposed to multiple forms of adverse childhood experiences are at greater risk of violent behaviour in adulthood and poor mental health outcomes. These include post-traumatic stress disorder, psychotic disorders, anxiety and mood disorders, and externalising disorders associated with violence, including conduct disorder, ASPD, drug and alcohol misuse. They also correspond to specific associations between childhood sexual abuse and perpetration of adult sexual violence.^29^

### Can associations with sexual violence be explained by non-sexual violence and psychopathology?

Because antisocial men and gang members were significantly more likely to show positive attitudes towards other violence, diagnoses of psychiatric morbidity and addictions, we investigated whether these factors explained their increased sexual violence and risk-taking. However, none of these explained the high levels of stalking, problem pornography use, anal sex, sex with prostitutes or multiple partners in the same encounter. This suggested these behaviours were either explained by other, unmeasured variables in adulthood or were more strongly associated with their earlier childhood maltreatment. However, the combination of positive attitudes towards violence, psychiatric morbidity and addictions did explain associations between gang membership and frequent attendance at strip/lap dancing clubs, partying and risk factors including infrequent use of contraceptives, being paid for sex, STIs and multiple sexual partners. The same combination of factors accounted for coercive sexual behaviour among the antisocial men group but not gang members, where other unmeasured factors or the persistence of early-onset sexually coercive behaviour were more important.

### Limitations

Our survey had several limitations, including the definition used to determine gang membership. However, there is no consensus about definition because gang structures have considerable heterogeneity. Nevertheless, we included three of the five UK criminal justice agency criteria.^19^

Quota sampling is the most commonly used non-probability sampling procedure in marketing research. Quota samples are collected to reflect proportions in the various subclasses or strata of the population of interest and the method is particularly useful when investigating hard-to-reach subgroups of the population and those who are likely to have a high rate of refusals to participate. Although our study samples matched the strata of the population of interest according to the national census (see supplementary material), we did not have a final figure for number of refusals to participate, as in a conventional survey, before the quotas in each subsample were met.

Although our study suggests violent and high-risk adult sexual behaviour had their origins in childhood, we did not have information on age when participants joined a gang. This meant that for some gang members, early sexual initiation and coercive sexual behaviour before age 15 could have been restricted to gang activity and not directly influenced by parental figures or early negative child care experiences. It is possible that some responders may overestimate some behaviours. However, self-report may have underestimated the true prevalence of their sexual behaviour because socially undesirable behaviours tend to be less frequently reported. We did not directly ask whether men had carried out acts of rape.

Diagnoses were also derived from self-report questionnaires and not confirmed by clinical interview, although self-report instruments can compare favourably with clinicians’ assessments. However, the community-based design and large sample size allowed us to examine associations between different categories of psychiatric morbidity and sexual behaviour, thus avoiding the selection bias associated with clinical samples. Furthermore, the sample size provided sufficient statistical power to test complex models and to control for confounding from demographic characteristics.

### Implications

**Fig. 1:**
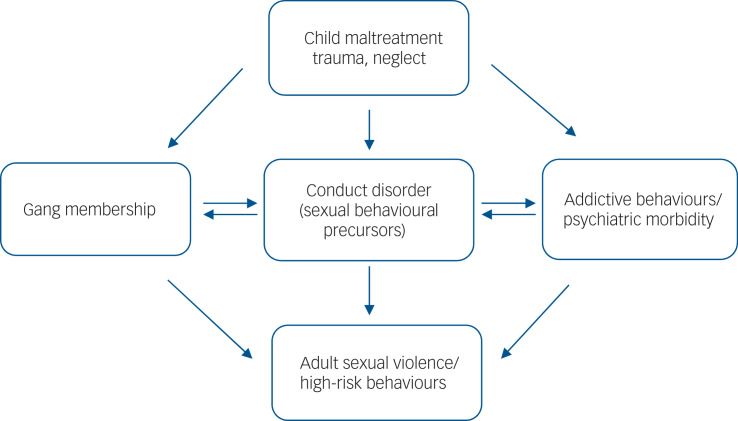
Hypothetical pathway model linking child maltreatment and adult sexual behaviours among gang members.

Street gangs should be recognised as a serious public health problem among socially excluded and minority youth in UK inner-urban areas characterised by socioeconomic deprivation, high crime and multiple social problems. Our findings explain the high level of sexual victimisation observed among gang-affiliated women in these areas.^2–4^ They suggest a pathway model from young men's early physical, sexual and emotional trauma to both sexual and non-sexual victimisation of others, with precursors of their violence appearing during childhood and persisting into adulthood, accompanied by multiple forms of risk-taking behaviour for STIs and HIV (Fig. 1). In this proposed model, childhood maltreatment and trauma increase the risk of both joining a gang and psychiatric morbidity, including addictions in early adulthood, as well as the development of conduct disorder. However, these pathways require confirmation in longitudinal studies. Nevertheless, our model of sexual violence corresponds to ‘cascading’ factors applied to non-sexual violence in gangs,^30^ with trajectories from problem behaviour in childhood, peer rejection and academic failure, subsequent deviant peer clustering, with deviancy training and violence during early adolescence, leading to more serious forms of violence in late adolescence. In this context, gang membership provides a peer group where all forms of violence are positively regarded that shapes roles, norms and expectations surrounding sex and sexual relationships, socialising expectations of masculine sexual prowess and female availability, and providing role models of masculinity.^24–26^ Gang membership provides potential victims for predation together with multiple opportunities and outlets for high-risk behaviour for older members, some of whom may be predisposed by damaging effects of early trauma on their subsequent psychosexual development and who sexually initiate new members.

The constellation of high-risk behaviours we have described, including sex with multiple partners, sex with men, sex workers, and more than one partner in the same encounter, anal sex, sex usually when intoxicated, and failure to use condoms, are well-recognised as increasing risks for STIs and HIV. This has received recognition as a public health problem in the USA^23,24^ but has not previously received attention in the UK. Our findings indicate that consideration should be given to educational programmes for gang members and affiliates, alerting them to these risks and to encourage screening to improve prevention and to reduce risk of infection being spread unknowingly within communities with high levels of gang activity.

Multiple addictions, including alcohol, drugs, gambling, viewing pornography, together with repetitive sexual behaviours indicative of hypersexuality and compulsion, with preference for forcing sex on non-consenting partners, may have determined persistence rather than desistence from gang membership for an older subgroup of men. US research has also described older gang members encouraging, organising and watching sexual behaviour among gang initiates, some of whom are legally still children, and where use of condoms is deliberately discouraged.^25^ Hypersexual behaviour with features of compulsion has been described as both an impulse disorder and behavioural addiction^31^ but was not included in DSM-5. Nevertheless, excessive engagement in behaviours such as gambling and sex share the same clinical, genetic, neurobiological and pharmacological parallels with substance addictions.^31,32^

Gangs in major UK cities have evolved since our survey with development of a ‘county lines’ business model of drug sales and supply from inner-urban areas where the markets have become saturated to rural areas and coastal towns to reach a new client base. Vulnerable children are targeted and groomed to distribute drugs, together with vulnerable adults whose properties are taken over to store, manufacture and sell drugs in these new locations (‘cuckooing’). In this context, girls are trafficked between gangs and subjected to sexual violence. Vulnerable women with accommodation are targeted, particularly drug users, and a drug debt to a gang will be repaid through prostitution.^33^

Sexual violence and gangs are not randomly distributed in a population and an ecological model has been proposed that takes into account the complex interplay of individual, relationship, social, cultural and environmental factors that determine violence with the aim of taking a preventive approach.^34^ In this context, sexual violence and risk-taking should be considered components of a closely aggregated group of health-related behaviours and psychiatric morbidity among gang members that interact synergistically to exacerbate their negative effects on each other^35^ and that may have underlying common causes. Many have their origins in childhood trauma but are later bound together by being in a gang.

## Data Availability

Data will be shared after approval of a proposal and with a signed data access agreement.

## Supplementary material

For supplementary material accompanying this paper visit https://doi.org/10.1192/bjp.2020.69. click here to view supplementary material
