# Supplementary material for: Gang membership and sexual violence: associations with childhood maltreatment and psychiatric morbidity
Source: Br J Psychiatry. 2020 Oct;217(4):583–90. doi: 10.1192/bjp.2020.69 (PMC7525108; doi:10.1192/bjp.2020.69)
Supplement: Supplementary file 1 [file S0007125020000690sup.zip › S0007125020000690sup001.docx]

**Supplementary materials**

(b)

Figure S1. Adjusted Odds Ratios of Sexual Behaviour, Psychiatric Morbidity, Addictions and Violence among Antisocial Men and Gang Members

*Table S1. Testing Explanations for the links between Gang Membership, Violent Behaviour, Psychiatric Morbidity, Addictive and Adult Sexual Behaviour: Antisocial compared with other men*

| Sexual Behaviour | Baseline | | Violent Behaviour | | | Psychiatric Morbidity | | | Addictive behaviour | | | Final Model | | |
| --- | --- | --- | --- | --- | --- | --- | --- | --- | --- | --- | --- | --- | --- | --- |
|  | OR | 95% CI | OR | 95% CI | % | OR | 95% CI | % | IR | 95% CI | % | IR | 95% CI | % |
| Violent Criminal | | | | | | | | | | | | | | |
| Coercive sex preference | 2.37*** | 1.47–3.81 | 2.00* | 1.13-3.57 | 19.4 | 1.85* | 1.09-3.14 | 28.5 | 1.88* | 1.07-3.31 | 26.4 | 1.69 | 0.89-3.20 | 39 |
| Stalking | 4.62*** | 2.70-7.88 | 3.51*** | 1.88-6.55 | 18 | 4.20*** | 2.37-7.48 | 6.1 | 4.09*** | 2.22-7.52 | 8 | 3.91*** | 1.95-7.82 | 10.9 |
| Compulsive behaviours | | | | | | | | | | | | | | |
| Strip/lap dancing clubs (≥ 2 weekly) | 0.59 | 0.30-1.17 | -1 | -1 | -1 | -1 | -1 | -1 | -1 | -1 | -1 | -1 | -1 | -1 |
| Partying (≥ 2 weekly) | 1.18 | 0.86-1.62 | -1 | -1 | -1 | -1 | -1 | -1 | -1 | -1 | -1 | -1 | -1 | -1 |
| Problem pornography use | 8.73*** | 3.44-22.11 | 8.67*** | 3.36-22.37 | 0.3 | 8.30*** | 3.00-22.90 | 2.3 | 4.79** | 1.87-12.28 | 27.7 | 5.83** | 1.91-17.73 | 18.6 |
| HIV/STI Risk | | | | | | | | | | | | | | |
| Contraceptives rare/never | 1.66*** | 1.32-2.08 | 1.49** | 1.17-1.90 | 20.6 | 1.57*** | 1.24-1.98 | 10.8 | 1.48** | 1.16-1.89 | 22.8 | 1.37* | 1.06-1.78 | 37.3 |
| Anal sex | 2.80*** | 2.21-3.56 | 2.44*** | 1.87-3.18 | 13.4 | 2.56*** | 1.99-3.28 | 8.9 | 2.27*** | 1.74-2.94 | 20.6 | 2.04*** | 1.54-2.71 | 30.7 |
| Paid for sex | 4.40*** | 2.82-6.86 | 3.55*** | 2.22-5.68*** | 14.5 | 3.79*** | 2.32-6.19 | 10.1 | 3.42*** | 2.05-5.71 | 17 | 2.84*** | 1.66-4.84 | 29.7 |
| STI (ever) | 4.39*** | 3.31-5.83 | 2.84*** | 2.07-3.90 | 29.4 | 4.05*** | 3.01-5.45 | 5.4 | 3.54*** | 2.59-4.84 | 14.6 | 2.51*** | 1.76-3.58 | 37.8 |
| 10+ partners past year | 1.95** | 1.29-2.96 | 1.39 | 0.82-2.33 | 51 | 1.69* | 1.07-2.69 | 21.3 | 1.39 | 0.86-2.25 | 50.8 | 1.15 | 0.64-2.06 | 79.6 |
| Prostitutes (10+) | 3.22** | 1.31-7.96 | 2.91* | 1.12-7.57 | 8.8 | 2.48 | 0.96-6.37 | 22.5 | 1.85 | 0.62-5.48 | 47.5 | 1.75 | 0.60-5.15 | 52 |
| Sex usually intoxicated | 3.56*** | 2.80-4.52 | 2.60*** | 1.98-3.42 | 24.6 | 3.16*** | 2.44-4.09 | 9.3 | 2.53*** | 1.93-3.31 | 27 | 2.09*** | 1.54-2.83 | 42.1 |
| Multiple partners same encounter | 3.44*** | 2.76-4.29 | 2.70*** | 2.13-3.43 | 19.6 | 3.39*** | 2.69-4.28 | 1.1 | 2.99*** | 2.34-3.83 | 11.3 | 2.58*** | 1.97-3.38 | 23.2 |
| Sex with men | 1.51 | 0.87-2.60 | -1 | -1 | -1 | -1 | -1 | -1 | -1 | -1 | -1 | -1 | -1 | -1 |

-1= no difference to explain;*= p < 0.05;**= p < 0.01;***= p < 0.001

*Table S2. Testing Explanations for the links between Gang Membership, Violent Behaviour, Psychiatric Morbidity, Addictive and Adult Sexual Behaviour: Gang members compared with other men.*

| Sexual Behaviour | Baseline | | Violent Behaviour | | | Psychiatric Morbidity | | | Addictive behaviour | | | Final Model | | | |
| --- | --- | --- | --- | --- | --- | --- | --- | --- | --- | --- | --- | --- | --- | --- | --- |
|  | OR | 95% CI | OR | 95% CI | % | OR | 95% CI | % | OR | 95% CI | % | OR | 95% CI | % | |
| Violent/Criminal | | | | | | | | | | | | | | |  |
| Coercive sex preference | 12.63*** | 6.76-23.57 | 7.73*** | 3.50-17.07 | 19.4 | 6.57*** | 3.16-13.65 | 25.8 | 4.79** | 1.64-14.03 | 38.2 | 4.34* | 1.38-13.66 | 42.1 | |
| Stalking | 42.89*** | 25.15-73.17 | 20.56*** | 10.50-40.27 | 19.6 | 33.49*** | 18.46-60.78 | 6.6 | 19.23*** | 9.18-40.27 | 21.3 | 16.68*** | 7.41-37.56 | 25.1 | |
| Compulsive behaviours | | | | | | | | | | | | | | |  |
| Strip/lap dancing clubs (≥ 2 weekly) | 3.39*** | 1.84-6.23 | 1.73 | 0.67-4.49 | 55.1 | 1.78 | 0.85-3.74 | 52.5 | 0.89 | 0.24-3.32 | 109.4 | 0.47 | 0.07-3.32 | 161.9 | |
| Partying (≥ 2/weekly) | 5.24*** | 3.25-8.43 | 3.39*** | 1.82-6.30 | 26.3 | 4.00*** | 2.44-6.56 | 16.2 | 2.99** | 1.50-5.98 | 33.8 | 1.85 | 0.82-4.13 | 63 | |
| Problem pornography use | 51.03*** | 25.82-100.85 | 49.52*** | 20.09-122.04 | 0.8 | 36.86*** | 15.47-87.84 | 8.3 | 19.87*** | 7.86-50.25 | 24 | 28.89*** | 9.34-89.43 | 14.5 | |
| HIV/STI Risk | | | | | | | | | | | | | | |  |
| Contraceptive rare/never | 2.80*** | 1.80-4.38 | 1.98* | 1.10-3.57 | 33.5 | 2.30** | 1.44-3.68 | 19.2 | 1.81 | 0.96-3.41 | 42.5 | 1.57 | 0.77-3.17 | 56.4 | |
| Anal sex | 9.32*** | 5.80-14.97 | 5.79*** | 3.03-11.04 | 21.3 | 7.85*** | 4.63-13.31 | 7.7 | 5.63*** | 2.94-10.76 | 22.6 | 4.58*** | 2.22-9.46 | 31.8 | |
| Paid for sex | 10.36*** | 5.93-18.09 | 5.71*** | 2.60-12.55 | 25.5 | 6.25*** | 3.15-12.43 | 21.6 | 4.13** | 1.55-10.98 | 39.3 | 2.5 | 0.81-7.73 | 60.8 | |
| STI (ever) | 13.47*** | 8.16-22.22 | 4.05*** | 2.13-7.72 | 46.2 | 10.05*** | 5.97-16.93 | 11.2 | 5.07*** | 2.69-9.56 | 37.6 | 2.24 | 1.00-5.03 | 68.9 | |
| 10+ partners past year | 6.63*** | 3.77-11.66 | 1.86 | 0.79-4.38 | 67.2 | 4.51*** | 2.48-8.19 | 20.4 | 2.23 | 0.94-5.30 | 57.6 | 1.31 | 0.48-3.61 | 85.7 | |
| Prostitutes (10+) | 27.65*** | 12.96-58.99 | 19.36*** | 6.30-59.51 | 10.7 | 13.62*** | 5.44-34.05 | 21.3 | 6.17* | 1.40-27.26 | 45.2 | 6.56** | 1.64-26.14 | 43.4 | |
| Sex usually intoxicated | 9.11*** | 5.31-15.64 | 3.62*** | 1.90-6.88 | 41.8 | 6.01*** | 3.47-10.41 | 18.8 | 2.21* | 1.12-4.39 | 64 | 1.51 | 0.68-3.36 | 81.4 | |
| Multiple partners same encounter | 10.16*** | 6.20-16.64 | 5.83*** | 3.17-10.69 | 24 | 9.14*** | 5.39-15.50 | 4.5 | 6.55*** | 3.45-12.44 | 18.9 | 4.53*** | 2.18-9.41 | 34.9 | |
| Sex with men | 7.65*** | 4.22-13.88 | 3.86* | 1.57-9.49 | 33.7 | 4.27*** | 2.32-7.87 | 28.7 | 3.73*** | 1.97-7.06 | 35.3 | 2.89** | 1.30-6.43 | 47.8 | |

-1= no explanatory variables meet criteria; *= p < 0.05; **= p < 0.01; ***= p < 0.001

*Table S3. Testing Explanations for links between Gang Membership, Violent Behaviours, Psychiatric Morbidity, Addictive, and Adult Sexual Behaviour: Gang members compared to Antisocial Men*

| Sexual Behaviour | Baseline | | Violent Behaviour | | | Psychiatric Morbidity | | | Addictive behaviour | | | Final Model | | |
| --- | --- | --- | --- | --- | --- | --- | --- | --- | --- | --- | --- | --- | --- | --- |
|  | OR | 95% CI | OR | 95% CI | % | OR | 95% CI | % | OR | 95% CI | % | OR | 95% CI | % |
| Violent/Criminal | | | | | | | | | | | | | | |
| Coercive sex preference | 5.33*** | 2.59-10.99 | 3.86*** | 1.83-8.11 | 19.3 | 3.55** | 1.61-7.83 | 24.4 | 2.54 | 0.95-6.82 | 44.3 | 2.57 | 0.94-7.04 | 43.7 |
| Stalking | 9.29*** | 4.89-17.66 | 5.86*** | 3.05-11.27 | 20.6 | 7.97*** | 4.13-15.38 | 6.9 | 4.71*** | 2.26-9.81 | 30.5 | 4.27*** | 2.05-8.97 | 34.9 |
| Compulsive behaviours | | | | | | | | | | | | | | |
| Strip/lap dancing clubs (≥ 2 weekly) | 5.70*** | 2.28-14.26 | 3.44* | 1.14-10.36 | 29.1 | 4.20* | 1.63-10.83 | 17.6 | 1.84 | 0.49-6.93 | 64.9 | 1.29 | 0.25-6.58 | 85.6 |
| Partying (≥ 2/weekly) | 4.44*** | 2.60-7.57 | 3.31*** | 1.78-6.16 | 19.7 | 3.83*** | 2.22-6.61 | 9.8 | 3.19** | 1.78-6.16 | 22.1 | 2.37* | 1.11-5.06 | 42 |
| Problem pornography use | 5.85*** | 2.10-16.31 | 5.71** | 1.91-17.08 | 1.3 | 4.44** | 1.48-13.32 | 15.6 | 3.39* | 1.16-9.91 | 30.9 | 4.08* | 1.24-13.37 | 20.4 |
| HIV/STI Risk | | | | | | | | | | | | | | |
| Contraceptive rare/never | 1.69* | 1.04-2.75 | 1.33 | 0.74-2.37 | 46 | 1.46 | 0.88-2.42 | 27.4 | 1.24 | 0.67-2.29 | 59.7 | 1.15 | 0.59-2.24 | 73.9 |
| Anal sex | 3.33*** | 1.99-5.55 | 2.37** | 1.27-4.42 | 28.2 | 3.07*** | 1.79-5.28 | 6.6 | 2.48** | 1.32-4.67 | 24.3 | 2.24* | 1.13-4.45 | 32.8 |
| Paid for sex | 2.35* | 1.22-4.54 | 1.61 | 0.74-3.51 | 44.4 | 1.64 | 0.81-3.34 | 42.2 | 1.21 | 0.48-3.03 | 78 | 0.88 | 0.31-2.49 | 114.5 |
| STI (ever) | 3.07*** | 1.77-5.32 | 1.43 | 0.75-2.73 | 68.3 | 2.48** | 1.42-4.34 | 18.9 | 1.43 | 0.76-2.71 | 67.9 | 0.89 | 0.42-1.92 | 110 |
| 10+ partners past year | 3.40*** | 1.85-6.23 | 1.34 | 0.65-2.77 | 76.1 | 2.58** | 1.38-4.81 | 22.6 | 1.6 | 0.73-3.53 | 61.4 | 1.12 | 0.47-2.66 | 91 |
| Prostitutes (10+) | 8.58*** | 3.29-225 | 6.65** | 2.26-19.62 | 11.8 | 5.50** | 1.97-15.37 | 20.7 | 3.34 | 0.89-12.46 | 43.9 | 3.74* | 1.06-13.21 | 38.6 |
| Sex usually intoxicated | 2.56** | 1.46-4.50 | 1.39 | 0.73-2.64 | 65.1 | 1.89* | 1.07-3.36 | 32 | 0.88 | 0.44-1.76 | 114.1 | 0.73 | 0.33-1.60 | 133.4 |
| Multiple partners same encounter | 2.95*** | 1.75-4.98 | 2.16* | 1.19-3.92 | 29 | 2.69*** | 1.56-4.63 | 8.7 | 2.19* | 1.18-4.07 | 27.6 | 1.76 | 0.88-3.49 | 48 |
| Sex with men | 5.07*** | 2.29-11.24 | 3.08* | 1.30-7.33 | 30.7 | 3.85** | 1.73-8.59 | 17 | 3.19** | 1.39-7.36 | 28.5 | 2.83* | 1.18-6.81 | 36 |

-^1^= no explanatory variables meet criteria; *= p < 0.05; **= p < 0.01; ***= p < 0.001

| *Supplementary Table S4. Patterns of missing values on Youth Male Survey sample* | | |
| --- | --- | --- |
|  | Missing frequency | Missing % |
| **Demographics** |  |  |
| Non-UK born | 75 | 1.61 |
| Being single | 41 | 0.88 |
| Unemployed | 110 | 2.36 |
| Ethnicity | 7 | 0.15 |
| Survey type | 0 | 0 |
| Age | 0 | 0 |
| **Sexual behaviour – violent / criminal** |  |  |
| Coercive sex (past year) | 583 | 12.52 |
| Coercive sex preference | 583 | 12.52 |
| Stalking | 39 | 0.84 |
| Conviction sex offence | 0 | 0 |
| **Sexual behaviour – compulsive behaviours** |  |  |
| Strip/lap dancing clubs (≥ 2 weekly) | 199 | 4.27 |
| Partying (≥2 weekly) | 117 | 2.51 |
| Problem pornography use | 121 | 2.60 |
| **Sexual behaviour – HIV/STI Risk** |  |  |
| Contraceptives rare/never | 619 | 13.30 |
| Anal sex | 340 | 7.30 |
| Paid for sex | 287 | 6.17 |
| STI (ever) | 302 | 6.49 |
| 10+ partners past year | 498 | 10.70 |
| Prostitutes (10+) ever | 269 | 5.78 |
| Sex usually intoxicated | 625 | 13.43 |
| Multiple partners (same encounter) | 326 | 7.00 |
| Sex with men | 283 | 6.08 |
| **Child Maltreatment** |  |  |
| Witnessing violence in home | 498 | 10.70 |
| Sexual abuse | 108 | 2.32 |
| Physical abuse | 285 | 6.12 |
| Neglect | 235 | 5.05 |
| First intercourse <13 years | 1,070 | 22.99 |
| In care | 160 | 3.44 |
| **Violent behaviour (past 5 years)** |  |  |
| Instrumental violence | 60 | 1.29 |
| Violence for excitement | 68 | 1.46 |
| Violent if disrespect | 553 | 11.88 |
| **Psychiatric morbidity** |  |  |
| Psychosis | 84 | 1.80 |
| Anxiety disorder | 52 | 1.12 |
| Depressive disorder | 56 | 1.20 |
| **Personality disorder** |  |  |
| Conduct disorder | 65 | 1.40 |
| Adult Antisocial (AAS) | 185 | 3.97 |
| Antisocial Personality Disorder | 106 | 2.28 |
| **Addictive behaviour** |  |  |
| Drug dependence | 105 | 2.26 |
| Alcohol dependence | 113 | 2.43 |
| Pathological gambling | 530 | 11.39 |

*Supplementary text: Quota Sampling and the 2011 Young Men's Health Survey - Report from ICM.*

Individuals were recruited by proportional quota sampling. This is a standard method that entails setting quotas for participants on a range of demographic factors and ensures that the sample interviewed is representative of the population of interest. It is particularly useful when investigating hard-to-reach samples, sensitive subjects such as violence, sexual behaviour, etc, and where it is anticipated that certain sub-sections of the population are less likely to complete interviews or questionnaires (e.g. young, male, lower social class, ethnic minorities, in areas of socioeconomic deprivation).

Quota sampling offers an alternative to probability sampling and is often used in market research and national surveys and becomes necessary if there is no listing of all those eligible to be included. It is more efficient as recruitment and sampling can be focused in areas in which the desired population are resident but does require good census data on the characteristics by which the quota are set. This method is preferred if the costs of probability sampling would be prohibitive and where feasibility issues become prohibitive.

Information on number of questionnaires returned was not collected because it is not a requirement of the methodology used here. The 2011 Young Men's Health Survey – and previous waves of research – adopted an in-home ‘random location’ with respondent self-completion and interviewer pick-up. Random location is the most common form for social and public policy research surveys as it combines a rigorous methodology with relatively low costs.

In this particular survey, we used Output Areas as the principal sampling unit. OAs are the base unit of the Census outputs and are based on groups of postcodes that fit within the boundaries of electoral wards/divisions and parishes. OAs represent the lowest geographical level on which full information can be generated through Census output. Each OA contains approximately 150 households and interviewers were required to achieve a target of 13 interviews (i.e. questionnaire pick-ups) in each OA.

A quota sheet was provided for each selected grouped OA, which reflects the actual composition of OA residents according to standard demographic criteria. Interviewers were required to interview a sample profile that exactly matches that of the grouped OA population profile using Census population information. This ensures that the sample is demographically representative at the micro-level, as well as geographically representative.

Crucially, interviewers were not required to record the number of questionnaires handed out, meaning it is not possible to record a response rate. This is in contrast to a random probability (pre-selected) survey where interviewers are required to visit randomly selected households and interview randomly selected individuals. With this technique, the response rate is recorded and taken as a measure of quality and robustness of the sample.

However, it is possible to work out an indicative response rate to the 2011 Young Men's Health Survey. ICM interviewers are required to leave a minimum of three addresses between calls, as well as include evening and weekend visits so that they maximise the chance of meeting their quotas and to avoid clustering in a single street. As such, it is reasonable to assume that interviewers distributed questionnaires to no more than a quarter of the households in each OA. This being the case, an approximate response rate would be around 35% (i.e. 13 completed surveys per point from 37.5 households visited). Nonetheless, this is an approximation and cannot be regarded as a true response rate.

RIM (Random Iterative Method) weighting is a technique commonly used to weight market research data to known targets, eg age groups regions, gender, and specifically to each variable (question) independently. The technique allows the analyst to adjust multiple characteristics in the dataset all at the same time in a way that ultimately keeps the different characteristics proportionate as a whole.

RIM weighting is a special form of target weighting. It can be a practical tool to use when there are targets (or populations) to which we wish the data for two or more variables, but not targets for the interlocking cells for these two or more variables. These are known as ‘rim weighting targets’. There may be more than two variables, which is where rim weighting is likely to be the chosen method. RIM weighting works by what is known as an iterative target weighting process. In other words, the software will calculate targets for the first rim and, after applying this weighting factor, it is highly improbable that the precise target percentages for one variable (eg gender) would be achieved. As the programme performs the iterations, the data gets closer and closer to the targets.

ICM uses Quantum, one of the most widely used tabulation and data packages in the survey research industry.

Translation by interviewers was not allowed which meant that to complete questionnaires the participants had to be English speakers.

Information on missing data from questionnaires is given in supplementary Table S4.

*Author contribution*

SU, CK and JC designed the study. CK and SU were responsible for data collection and cleaning. CK, RG and YYL performed the analyses and all authors including JW, ZQ, and YZ contributed to the interpretation of results. JC drafted the manuscript which all authors commented on and approved for submission.
